# Facile Fabrication of Reduction-Responsive Supramolecular Nanoassemblies for Co-delivery of Doxorubicin and Sorafenib toward Hepatoma Cells

**DOI:** 10.3389/fphar.2018.00061

**Published:** 2018-02-06

**Authors:** Qingqing Xiong, Mangmang Cui, Ge Yu, Jian Wang, Tianqiang Song

**Affiliations:** ^1^Department of Hepatobiliary Cancer, National Clinical Research Center for Cancer, Key Laboratory of Cancer Prevention and Therapy, Tianjin's Clinical Research Center for Cancer, Tianjin Medical University Cancer Institute and Hospital, Tianjin, China; ^2^Hebei province Cangzhou Hospital of Integrated Traditional and Western Medicine, Cangzhou, China; ^3^Department of Immunology, National Clinical Research Center for Cancer, Key Laboratory of Cancer Prevention and Therapy, Tianjin's Clinical Research Center for Cancer, Tianjin Medical University Cancer Institute and Hospital, Tianjin, China

**Keywords:** reduction-responsive, supramolecular nanoassemblies, doxorubicin, sorafenib, hepatocellular carcinoma

## Abstract

Combination of doxorubicin with sorafenib (SF) was reported to be a promising strategy for treating hepatocellular carcinoma (HCC). In this study, we designed a reduction-responsive supramolecular nanosystem based on poly (ethylene glycol)-β-cyclodextrin (PEG-CD) and a disulfide-containing adamantine-terminated doxorubicin prodrug (AD) for efficient co-delivery of doxorubicin and sorafenib. PEG-CD/AD supramolecular amphiphiles were formed through host-guest interaction between cyclodextrin and adamantine moieties, and then self-assembled into regular spherical nanoparticles with a uniform size of 166.4 nm. Flow cytometry analysis and confocal laser scanning microscopy images showed that PEG-CD/AD nanoparticles could be successfully taken up by HepG2 cells and then released doxorubicin into the cell nuclei. Moreover, sorafenib could be facilely encapsulated into the hydrophobic cores to form PEG-CD/AD/SF nanoparticles with a slightly larger size of 186.2 nm. PEG-CD/AD/SF nanoparticles sequentially released sorafenib and doxorubicin in a reduction-response manner. *In vitro* cytotoxicity assay showed that PEG-CD/AD/SF nanoparticles had an approximately 4.7-fold decrease in the IC_50_ value compared to that of PEG-CD/AD and SF physical mixtures, indicating stronger inhibitory effect against HepG2 cells by co-loading these two drugs. In summary, this novel supramolecular nanosystem provided a simple strategy to co-deliver doxorubicin and sorafenib toward hepatoma cells, which showed promising potential for treatment of HCC.

## Introduction

Hepatocellular carcinoma (HCC) is the second leading cause of cancer-related death (International Agency for Research on Cancer. GLOBOCAN, 2012), characterized by its insidious onset, poor diagnosis, and intrinsic resistance to chemotherapy agents. Doxorubicin (DOX), a widely used chemotherapeutic drug, plays an undisputed key role in transcatheter arterial chemoembolization (TACE) for HCC (Vilaseca et al., 1978). However, the therapeutic efficacy of DOX for HCC is often limited because of the emergence of drug resistance (Deng et al., 2015; Galun et al., 2017) and its irreversible cardiotoxicity (Singal and Iliskovic, 1998). Sorafenib (SF), an oral multiple kinase inhibitor, is successful to prolong the survival of advanced HCC patients and has been approved by FDA for the standard treatment of patients with unresectable HCC (Llovet et al., 2015). However, during the clinical application, it was found that SF was only beneficial to about 33% HCC patients while showed low tumor response to other majority of patients (Cheng et al., 2009). It has been reported that combining two or more drugs of different molecular mechanisms might be a promising alternative option for long-term application of a single drug and would exert better therapeutic effects on HCC (Lin et al., 2016; Sun et al., 2017; Zhao et al., 2017). Moreover, clinical trial studies have demonstrated that combination of DOX with SF exhibited remarkable improvement in the overall survival of HCC patients (Hutchinson, 2011; Pazo Cid et al., 2011).

However, due to the hydrophobic properties and varied pharmacokinetic profiles of these two drugs, the co-delivery of both drugs toward HCC is still a major challenge. With the development of nanotechnology in biomedical fields, nanoparticles provide a valid platform to achieve co-loading of multiple drugs and deliver them toward tumor cells simultaneously (Hu et al., 2016). Among these nanoparticles, supramolecular nanosystems, especially formed by host-guest interaction, have attracted researchers' great attention in drug delivery (Feng et al., 2017; Ping et al., 2017). Cyclodextrins (CDs), a series of natural cyclic oligosaccharides composed of D-glucose units, are the most commonly used macrocyclic hosts to construct supramolecular nanosystems in virtue of their low toxicity and low immunogenicity (Zhang et al., 2013; Feng et al., 2017; Xiong et al., 2017). The most prominent structural feature of CDs is their hydrophobic cavities, which can accommodate a variety of guest molecules such as adamantine (Luo et al., 2012) and trans-azobenzene (Li et al., 2013) *via* host-gust interaction. During the past few decades, CDs and their derivatives have been extensively utilized in the construction of drug delivery system. Zhang and coworkers established a supramolecular photosensitizer delivery system through the self-assembly of supramolecular amphiphiles constructed by the host–guest interaction between poly (ethylene glycol)-β-cyclodextrin and adamantane-terminated porphyrin derivatives (Liu et al., 2015). In our previous study, a series of well-defined cyclodextrin-based polymers were synthesized by atom transfer radical polymerization and then further used for drug delivery (Xiong et al., 2014; Zhang M. et al., 2014).

Recently, stimuli-responsive nanoparticles have drawn a lot of attentions due to their responsiveness to tumor or intracellular micro-environmental stimuli, such as low pH-values (Chen et al., 2016), high concentration of certain enzymes (Zhang et al., 2017), and more reductive environment (Wang et al., 2013), and thus releasing their payloads in a controlled manner. According to the previous studies (Wang et al., 2012; Yin et al., 2013), the cytosolic glutathione (GSH) concentration is around 2–10 mM, which is substantially higher than that in the extracellular fluids and blood (2–10 μM). Disulfide bond, which is stable in blood circulation but can be cleaved under reducing intracellular microenvironment, has been widely used as a linker in the design of reduction-responsive drug delivery system. In Sun's work, they reported a prodrug-based nanoplatform self-assembled by the disulfide bond linked conjugates of paclitaxel (PTX) and oleic acid, achieving the rapid and differential release of PTX in tumor cells (Luo et al., 2016). Moreover, the prodrug strategy provides several advantages such as enhanced *in vivo* stability, the prolonged half-life in blood, the improved water solubility, etc. (Yang et al., 2015; Li D. et al., 2016; Li W. et al., 2016).

**Figure 1:**
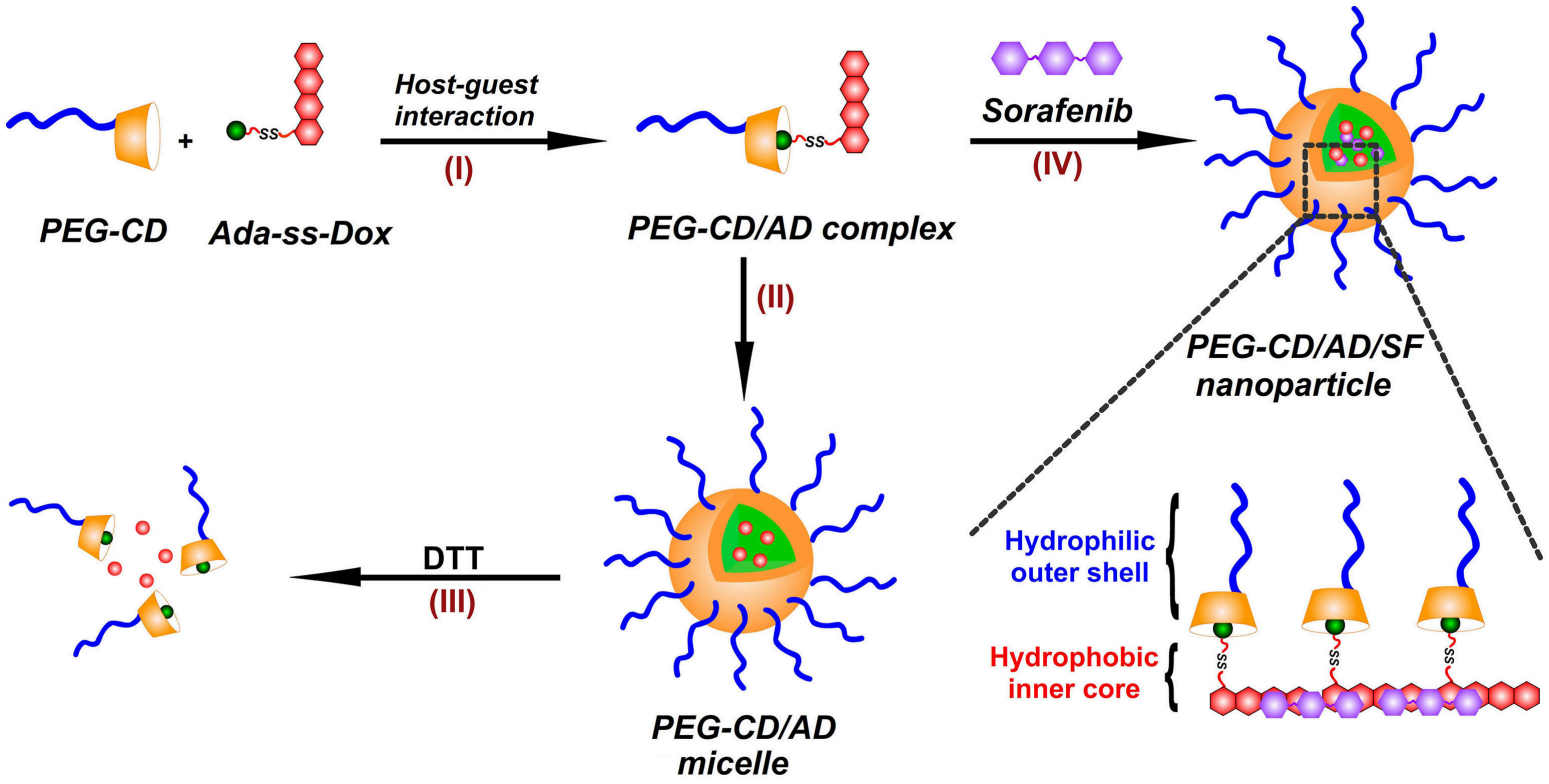
Illustration for the preparation of PEG-CD/AD/SF nanoparticles. (I) PEG-CD/AD supramolecular amphiphile was formed *via* the host-gust interaction between CD cavity and Ada moiety; (II) PEG-CD/AD nanoparticles were prepared by self-assembly of PEG-CD/AD amphiphile in aqueous medium; (III) PEG-CD/AD nanoparticles disassemble after incubation with DTT; (IV) PEG-CD/AD/SF nanoparticles were prepared by incorporating SF in the self-assembly process of PEG-CD/AD amphiphile.

In this paper, we prepared a novel reduction-responsive supramolecular nanosystem by a facile method for co-delivery of DOX and SF toward hepatoma cells (Figure 1). A reduction-responsive prodrug of doxorubicin (Ada-ss-DOX) was first synthesized by conjugation of doxorubicin with amantadine through disulfide bond, and then complexed with poly (ethylene glycol)-β-cyclodextrin (PEG-CD) *via* host-guest interaction between CD cavity and Ada moiety to form PEG-CD/AD supramolecular amphiphiles (I). Using a modified nanoprecipitation method, PEG-CD/AD amphiphiles could self-assemble into spherical nanoparticles in aqueous medium (II). The reduction-responsive disassembly of PEG-CD/AD nanoparticles triggered by dithiothreitol (DTT) was then investigated (III). Moreover, SF could also be incorporated into the hydrophobic cores of the nanoparticles by hydrophobic interaction and π-π stacking interaction with DOX (IV), and thus achieved the co-loading of DOX and SF. The size and morphology of these nanoparticles were respectively characterized by dynamic light scattering (DLS) and transmission electron microscopy (TEM). The cellular uptake and intracellular locations of PEG-CD/AD nanoparticles were investigated by flow cytometry analysis and confocal laser scanning microscopy (CLSM). In addition, the cytotoxicity of PEG-CD/AD/SF nanoparticles against hepatoma HepG2 cells were evaluated by CCK-8 assay in detail.

## Materials and methods

### Materials

Amantadine hydrochloride (Ada), and dithiodiglycolic acid (DTGA, ≥98%) 4′,6-diamidino-2-phenylindole (DAPI) were obtained from Sigma-Aldrich (St. Louis, MO, USA) and used without further purification. Doxorubicin hydrochloride (DOX·HCl) and sorafenib (SF) base was purchased from Dalian Meilun Biotech Co., Ltd. (Dalian, China). 1-(3-Dimethylaminopropyl)-3-ethylcarbodiimide hydrochloride (EDC·HCl), 1-hydroxybenzotriazole hydrate (HOBt, 99%), O-(7-azabenzotriazol-1-yl)-N,N,N′,N′-tetramethyluronium hexafluorophosphate (HATU, 99%), and N,N-diisopropylethylamine (DIPEA, 99%) was purchased from J&K Scientific (Beijing, China). Methoxypoly(ethylene glycol) succinimidyl succinate (PEG-NHS, M_n_ = 2000) was purchased from JenKem Technology Co., Ltd (Beijing, China). Other chemical reagents in the study were analytical grade and obtained from commercial sources. PEG-CD was synthesized according to the previous study (Peng et al., 2014) and the synthetic route was described in the supplementary data (Figure S1A). The ^1^H NMR spectrum of PEG-CD was displayed in Figure S1B.

### Synthesis of amantadine-terminated doxorubicin prodrug (Ada-ss-DOX)

Ada-ss-DOX was synthesized by the following two steps. DTGA (5 mmol, 0.9110 g) was dissolved 15 mL of dichloromethane. Subsequently, HOBt (4.5 mmol, 0.6070 g), EDC·HCl (4.5 mmol, 0.8612 g) and triethylamine (15 mmol, 1.5178 g) were added. After the mixture was stirred at 20°C for 1 h, Ada (3 mmol, 0.4549 g) was added. The resulting mixture was stirred at 20°C for another 3 h and filtered to give the filtrate. The filtrate was concentrated to give the crude product and then purified by prep-HPLC system (Instrument: GILSON 281, Column: ASB C18 150^*^25 mm. Mobile phase: A: deionized water containing 0.1% HCl; B: acetonitrile, Gradient: B from 40 to 70% in 10 min. Flow rate: 25 mL/min) to obtain Ada-ss-COOH as a brown solid (Yield 6.8%).

Subsequently, the synthesized Ada-ss-COOH (3.17 mmol, 1.00 g) and DOX·HCl (2.85 mmol, 1.65 g) were co-dissolved in 20 mL dimethyl formamide (DMF) and then HATU (4.76 mmol, 1.81 g) and DIPEA (9.51 mmol, 1.23 g) were added. The mixture was stirred at 20°C for 2 h and then diluted by 20 mL of water. The resulting mixture was extracted with ethyl acetate. The organic phases were collected, washed with brine and dried with anhydrous Na_2_SO_4_, filtered and finally concentrated under vacuum to give Ada-ss-DOX (hereafter “AD”) as a red solid powder (Yield 45.5%).

### Preparation of PEG-CD/AD and PEG-CD/AD/SF nanoparticles

PEG-CD/AD nanoparticles were prepared by a modified nanoprecipitation and dialysis method. PEG-CD and AD were dissolved in DMF respectively to obtain solutions with a concentration of 1 mg/mL. Then, PEG-CD solution and Ada-ss-DOX solution with molar ratio of 1:1 was mixed and stirred overnight to obtain PEG-CD/AD supramolecular amphiphiles. Afterwards, an equal volume of water was slowly dropped into the above mixed solution under stirring at 400 rpm. The solution was stirred for another 4 h, followed by dialyzed against deionized water for 24 h to remove DMF (MWCO 3500). The dialysate was further concentrated by ultra-filtration membrane (MWCO 5000).

PEG-CD/AD/SF nanoparticles were prepared by a similar method. A determined volume of SF solution in DMF was stirred with PEG-CD/AD mixed solution for 4 h and then water was added. The solution was stirred, dialyzed and concentrated as described above to obtain PEG-CD/AD/SF nanoparticles.

### Characterization

^1^H NMR analysis was carried out on a AVANCE III HD nuclear magnetic resonance spectrometer at 400 MHz (Bruker, Germany). The molecular weight of AD was analyzed by mass spectrometry (Agilent, 6510 Q-TOF LC/MS, USA). The size, size distribution, and zeta potential of the nanoparticles in aqueous solution were determined by a Zetasizer Nano ZS instrument (Malvern, England) at 25°C. The nanoparticle morphology was visualized using Tecnai G2 20STwin transmission electron microscope (FEI, USA).

### Reduction-triggered disassembly of PEG-CD/AD nanoparticles

PEG-CD/AD nanoparticles were added into DTT solutions with different concentrations and then treated for determined periods. The fluorescence intensities of the mixed solutions were measured on a F-4500 fluorescence spectrometer (Hitachi, Japan) at an excitation wavelength of 485 nm. Meanwhile, the size and morphology changes of PEG-CD/AD nanoparticles were monitored by DLS and TEM.

### Cellular uptake and intracellular distributions of PEG-CD/AD nanoparticles

Flow cytometry was employed to determine the cellular uptake of PEG-CD/AD nanoparticles in HepG2 cells. Briefly, the cells were seeded on a 12-well plate and cultured for 24 h. Then the cultural media were replaced with fresh media containing free DOX or PEG-CD/AD nanoparticles at a DOX concentration of 2 μg/mL. After incubation for determined periods, the cells were collected and analyzed on a BD FACSVerse™ flow cytometer (BD Biosciences, USA).

For confocal laser scanning microscope (CLSM) study, the cells were placed onto 12-well glass plates. After incubation for 24 h, the cells were treated as the same as flow cytometry analysis. Then the cells were washed with PBS, fixed with 4% formaldehyde and the cell nuclei were stained by DAPI. Thereafter, the cells were observed under FV 1000 CLSM (Olympus, Japan).

### *In vitro* drug releases of DOX and SF from PEG-CD/AD/SF nanoparticles

The *in vitro* releases of DOX and SF from PEG-CD/AD/SF nanoparticles were evaluated using dynamic dialysis method in four kinds of release media (I: pH 7.4 PBS; II: pH 7.4 PBS with 10 mM DTT; III: pH 5.0 PBS; IV: pH 5.0 PBS with 10 mM DTT). Briefly, PEG-CD/AD/SF nanoparticle solutions (1 mL) were placed into dialysis bags (MWCO 7000) and dialyzed against above media at 37°C in the air bath shaking with 100 rpm. At designated time points, 0.5 mL of release media was withdrawn and replaced with an equal volume of fresh release media. The amounts of DOX and SF in the release media were determined by UV/Vis spectrophotometer (Beckman DU-640, USA) at a wavelength of 490 and 267 nm, respectively (Malarvizhi et al., 2014). The release tests were repeated for three times and the accumulative releases of drug were calculated.

### *In vitro* cytotoxicity analysis

The cytotoxicities of PEG-CD/AD and PEG-CD/AD/SF nanoparticles compared with free DOX, free SF, and PEG-CD/AD nanoparticles and SF physical mixtures were evaluated in HepG2 cells using CCK-8 regents. Briefly, the cells were seeded onto 96-well plate and cultured for 24 h, and then the media were replaced with fresh media containing free DOX, free SF, PEG-CD/AD nanoparticles, PEG-CD/AD and free SF physical mixtures, and PEG-CD/AD/SF nanoparticles followed by further incubation for 48, 72, and 96 h, respectively. Next, the media were removed and 10 μL of CCK-8 regents and 90 μL of fresh media were added into each well. After incubated for 2 h, the plates were gently shaken for 2 min and the absorbance was measured at 450 nm using an Epoch 2 microplate spectrophotometer (BioTek, Winooski, USA). The cell viabilities were calculated as the ratio of the absorbance values of treated cells to those of untreated cells. The inhibitory effect of PEG-CD on HepG2 cell growth for 48 h was also assessed as the similar method.

### Statistical analysis

Each experiment was repeated for three times. All data were presented as mean ± standard deviation and compared using one-way ANOVA. The differences were significant when *p* < 0.05.

## Results and discussion

### Synthesis and characterization of Ada-ss-DOX

**Figure 2:**
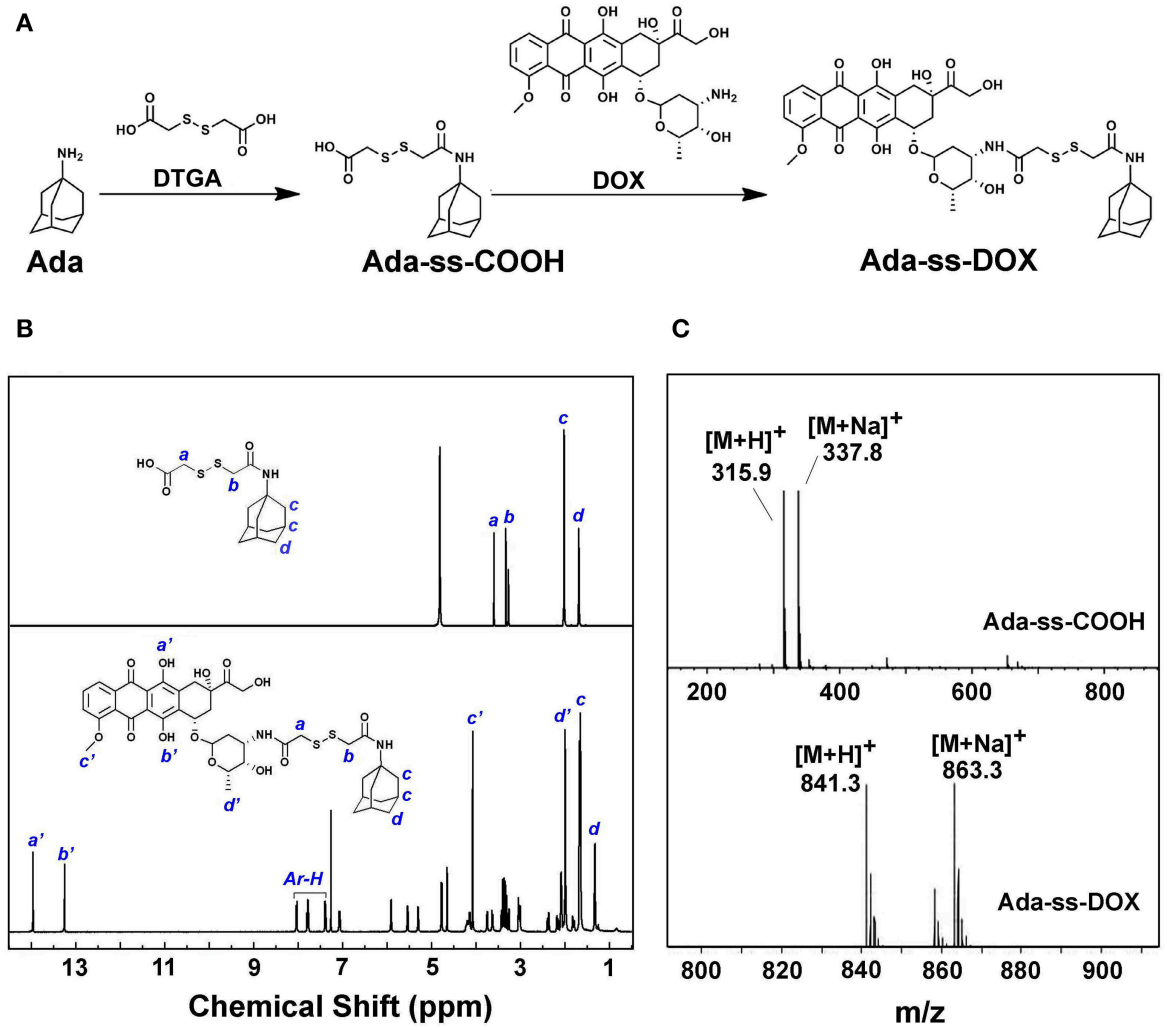
Synthetic route **(A)**, ^1^H NMR spectra **(B)** and mass spectra **(C)** of Ada-ss-DOX.

DTGA, with two active carboxyl groups, was chosen as the disulfide reagent to produce Ada-ss-DOX prodrug. The synthesis route is displayed in Figure 2A. Firstly, Ada-ss-COOH was synthesized *via* the condensation reaction between Ada and DTGA. To ensure that the product with only one of the carboxyl groups of DTGA conjugated with Ada was collected, excess DTGA was added during the reaction process and the product was further purified by prep-HPLC. From the ^1^H NMR spectrum of Ada-ss-COOH in Figure 2B, the integration ratio of the peaks at 3.66 and 3.38 ppm, corresponding to the methylene protons of DTGA (*signal a and b*), to the peaks at 1.75 ppm, originating from the methylene protons of Ada (*signal d*), was calculated to be 1:3, which indicated that the molar ratio of Ada and DTPA is 1:1. Moreover, the molecular weight of Ada-ss-COOH (C_14_H_22_NO_3_${\text{S}}_{2}^{+}$) determined by ESI-MS in Figure 2C was found to be 315.9 g/mol (calcd 316.5 g/mol), further indicating the success synthesis of Ada-ss-COOH.

Subsequently, Ada-ss-DOX was obtained by the reaction between the end carboxyl group of Ada-ss-COOH and the reactive amine group of DOX. The characteristic peaks of hydroxyl protons of DOX (*signal a' and b'*) located at 13.93 and 13.20 ppm were obviously observed in the ^1^H NMR spectrum of Ada-ss-DOX (Figure 2B). Due to the steric-hindrance effect after conjugation of DOX, the signals attributed to the methylene protons of DTGA (*signal a and b*) significantly decreased in the^1^H NMR spectrum of Ads-ss-DOX. The ESI-MS result showed that the molecular weight of Ada-ss-DOX (C_41_H_49_N_2_O_13_${\text{S}}_{2}^{+}$) was 841.3 g/mol, which was consistence with calculated 841.9 g/mol (Figure 2C). Altogether, the above results demonstrated that Ada-ss-DOX was successfully prepared.

### Preparation and characterization of PEG-CD/AD nanoparticles

**Figure 3:**
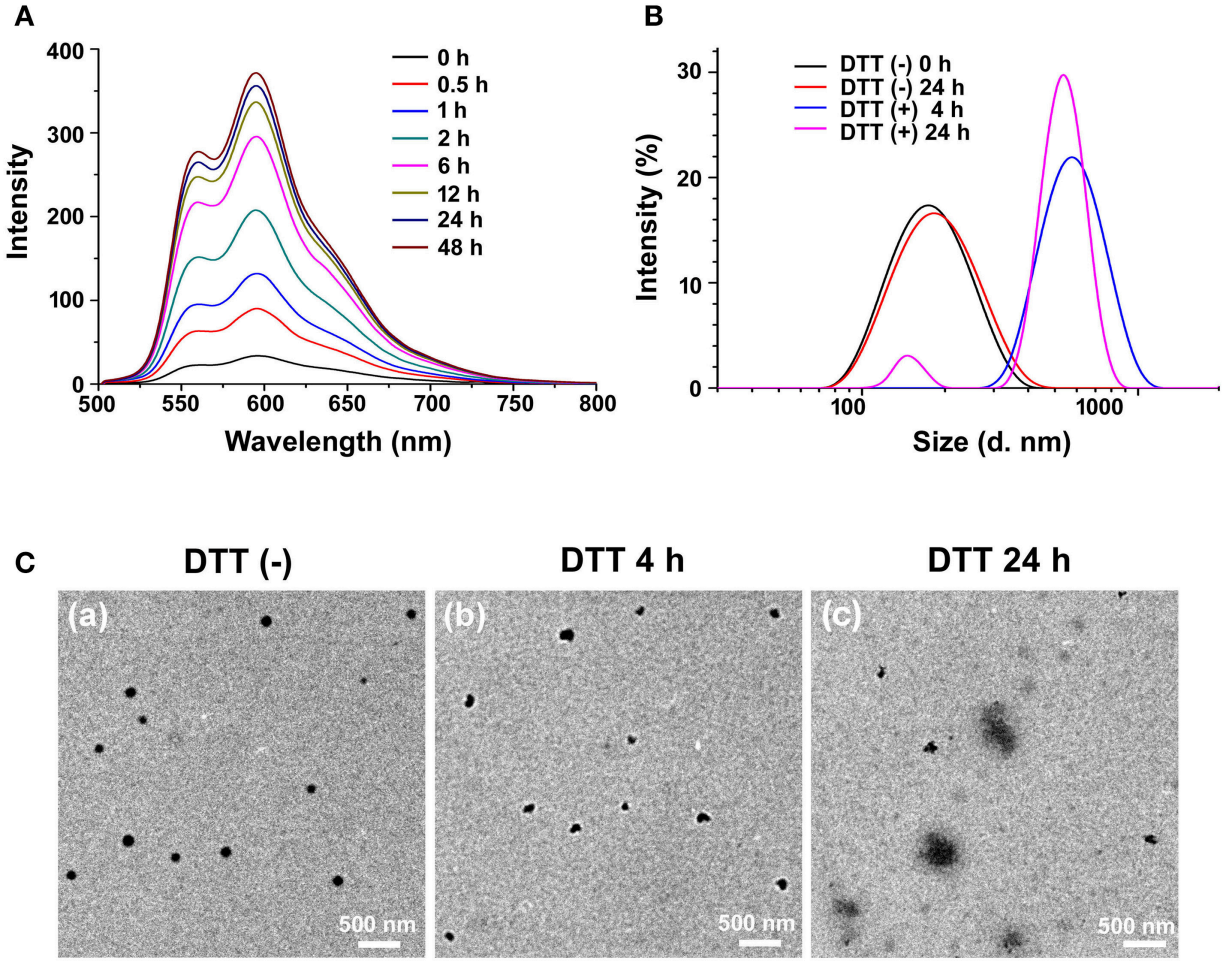
Reduction-responsive disassembly of PEG-CD/AD nanoparticles triggered by DTT. **(A)** Emission spectra of DOX in PEG-CD/AD solutions containing DTT at different time with an excitation wavelength of 485 nm. [PEG-CD/AD = 40 μg/mL] **(B)** Hydrodynamic diameter distribution change of PEG-CD/AD nanoparticles treated with 10 mM DTT for 24 h; **(C)** TEM images of PEG-CD/AD nanoparticles treated without DTT **(a)** and with 10 mM DTT for 4 h **(b)** or 24 h **(c)**.

To our knowledge, the formation of nanoparticles was often due to the self-assembly process of amphiphilic block copolymers driven by hydrophobic interaction among the hydrophobic segments (Zhao et al., 2012). In this study, PEG-CD/AD nanoparticles were self-assembled from PEG-CD/AD supramolecular amphiphiles with hydrophilic segment PEG-CD and hydrophobic segment DOX (Figure 1). The DMF solution of PEG-CD and AD was stirred overnight to obtain the supramolecular amphiphiles *via* host-guest interaction between CD cavities and Ada moieties, and then the deionized water was slowly added to induce the self-assembly of PEG-CD/AD nanoparticles. Finally, the solution was dialyzed against deionized water to remove DMF. The DOX content was calculated to be 13.5% as the molar ratio of PEG-CD and AD was 1:1. The hydrodynamic diameter and morphology of PEG-CD/AD nanoparticles was determined by DLS and TEM. As shown in Figure 3B, the average size of PEG-CD/AD nanoparticles was 166.4 nm and polydispersity (PDI) of 0.089. TEM image in Figure 3Ca showed that PEG-CD/AD nanoparticles had spherical shape and the average particle size was about 100 nm, which was significantly smaller that measured by DLS. It was perhaps because that the PEG chains in the outer surface of PEG-CD/AD nanoparticles were extended in aqueous solution while collapsed in dry samples.

### Reduction-responsive disassembly of PEG-CD/AD nanoparticles

DTT was used to mimic the reductive environment as it was a prevailing GSH stimulant (Dai et al., 2011; Zhang B. et al., 2014). As shown in Figure 3A, the fluorescence intensity of the PEG-CD/AD solution containing 10 mM DTT gradually increased as time prolonged. By contrast, almost no change in the emission spectra of the PEG-CD/AD solution was observed for 48 h in the absence of DTT (Figure S2). Moreover, the emission spectra of the nanoparticles were also detected after incubation with DTT of different concentrations for 2 h. It was obviously found that the fluorescence intensity increased with the DTT concentration increasing from 1 to 50 mM (Figure S3). The above results suggested that the disulfide bond could be cleaved under the stimulation of DTT, resulting in the disassembly of PEG-CD/AD nanoparticles and release of DOX.

The hydrodynamic diameters of PEG-CD/AD nanoparticles were also measured after incubation with DTT for 4 h and 24 h (Figure 3B). After 4 h, the average size of the nanoparticles increased to 683.6 nm with a corresponding increase of PDI to 0.29. The nanoparticles changed to be larger than 1 μm with PDI of 0.89 after 24 h incubation. TEM images in Figures 3Cb,**c** showed that the morphology of the nanoparticles was irregular and large aggregated particles could be observed, which further confirmed that the disassembly due to reductive cleavage of the disulfide bonds. To be noticed, the hydrodynamic diameters of PEG-CD/AD nanoparticles after incubation with DTT seemed larger than the sizes of nanoparticles displayed in TEM images. This might be attributed that the average size measured by DLS was determined by intensity, and in this case, the size would be larger than the actual size when very large aggregated particles existed.

### Cellular uptake and intracellular locations of PEG-CD/AD nanoparticles

**Figure 4:**
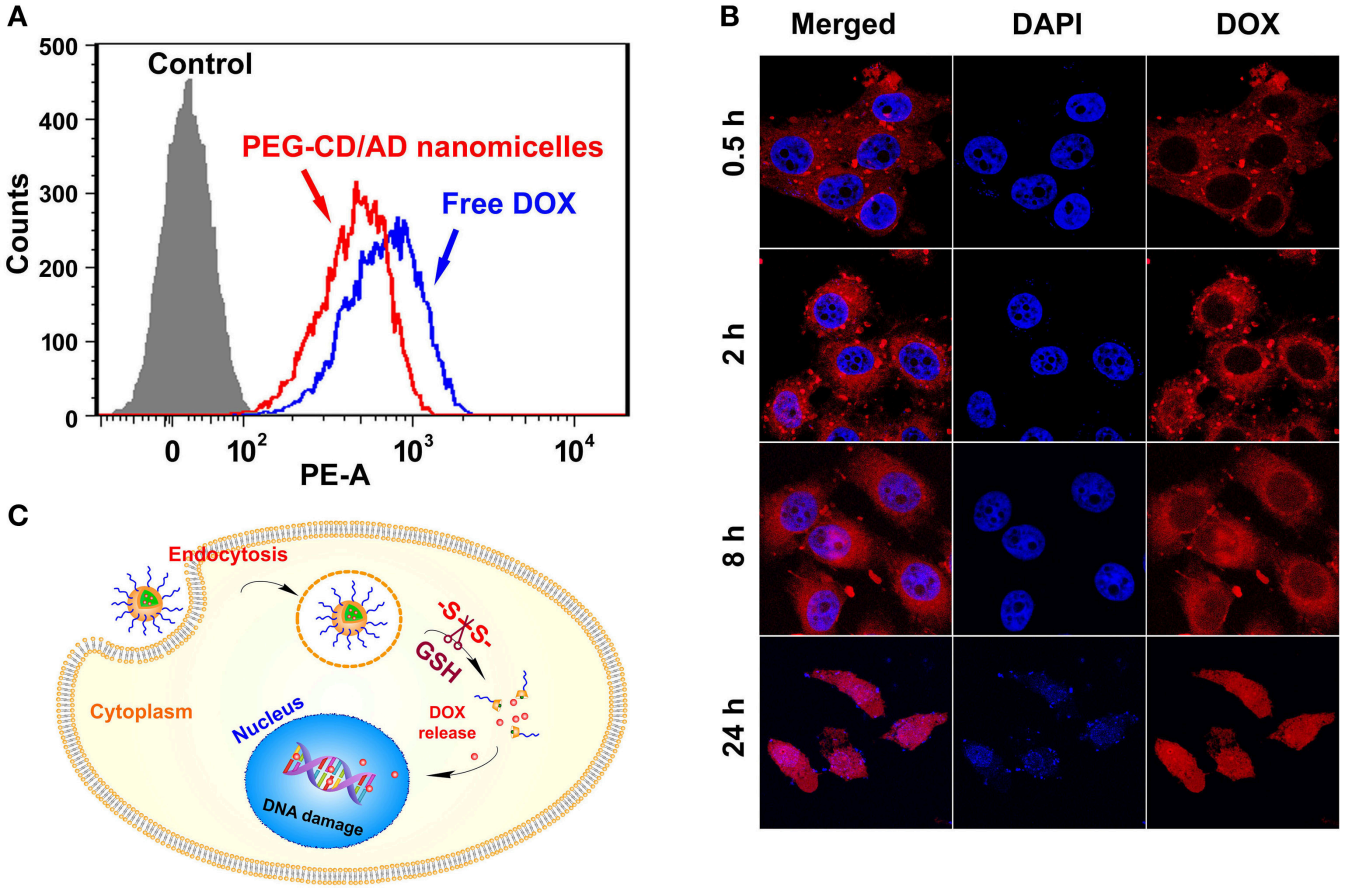
Cellular uptake and intracellular locations of PEG-CD/AD nanoparticles in HepG2 cells. **(A)** Flow cytometry analysis of the cells with the treatments of PEG-CD/AD nanoparticles for different time. **(B)** Confocal images of the cells. **(C)** Schematic illustration of cell internalization of PEG-CD/AD nanoparticles and intracellular release of DOX.

The cellular uptake of PEG-CD/AD nanoparticles was investigated in HepG2 cells by flow cytometry and CLSM. HepG2 cells were incubated with PEG-CD/AD nanoparticles or free DOX for 2 h at DOX concentration of 2.0 μg/mL and then collected for flow cytometry analysis. As shown in Figure 4A, PEG-CD/AD nanoparticles exhibited much stronger fluorescence intensity than the control, which indicated that the nanoparticles could be effectively internalized into HepG2 cells. However, PEG-CD/AD nanoparticles showed slightly lower intracellular fluorescence intensity than that of free DOX. It was probably because DOX could not be completely released from the nanoparticles in 2 h.

Next, CLSM was employed to track the intracellular locations of PEG-CD/AD nanoparticles after internalization. HepG2 cells were treated with PEG-CD/AD nanoparticles for predetermined time and the nuclei were stained by DAPI (blue). As shown in Figure 4B, the fluorescence signals of DOX (red) in the cells exhibited time-dependent manner. Weak fluorescence signals were observed in the cytoplasm after 0.5 h of incubation and the signals tended to be stronger after 2 h of incubation. Minor red fluorescence signals could be observed in the nuclei after 8 h of incubation, suggesting that DOX was released from the nanoparticles and began to accumulate in the nuclei. After 24 h, the red fluorescence signals were almost overlapped with the shrunken nuclei, which indicated that most of DOX entered the nuclei and exerted an evident cytotoxic effect. Besides, the dynamic intracellular process of free DOX was also investigated and the results are shown in Figure S4. Unlike PEG-CD/AD nanoparticles, free DOX could rapidly enter the nuclei since the red fluorescence signals were obviously observed in the nuclei after 0.5 h. The drug release from free DOX was much faster than that from DOX-loaded nanoparticles (Figure S5), thus, this difference might be attributed to the fact that free DOX can quickly transport into the cells *via* passive diffusion, while PEG-CD/AD nanoparticles entered the cells through endocytosis and then released DOX into the nuclei, thus showing a lagged effect. According to the above results, we might draw the conclusion that, after PEG-CD/AD nanoparticles were taken up, the disulfide bond could be cleaved by the high GSH environment in the cytoplasm and PEG-CD/AD nanoparticles disassembled to release free DOX (Figure 4C).

### *In vitro* cytotoxicity of PEG-CD/AD nanoparticles against HepG2 cells

**Figure 5:**
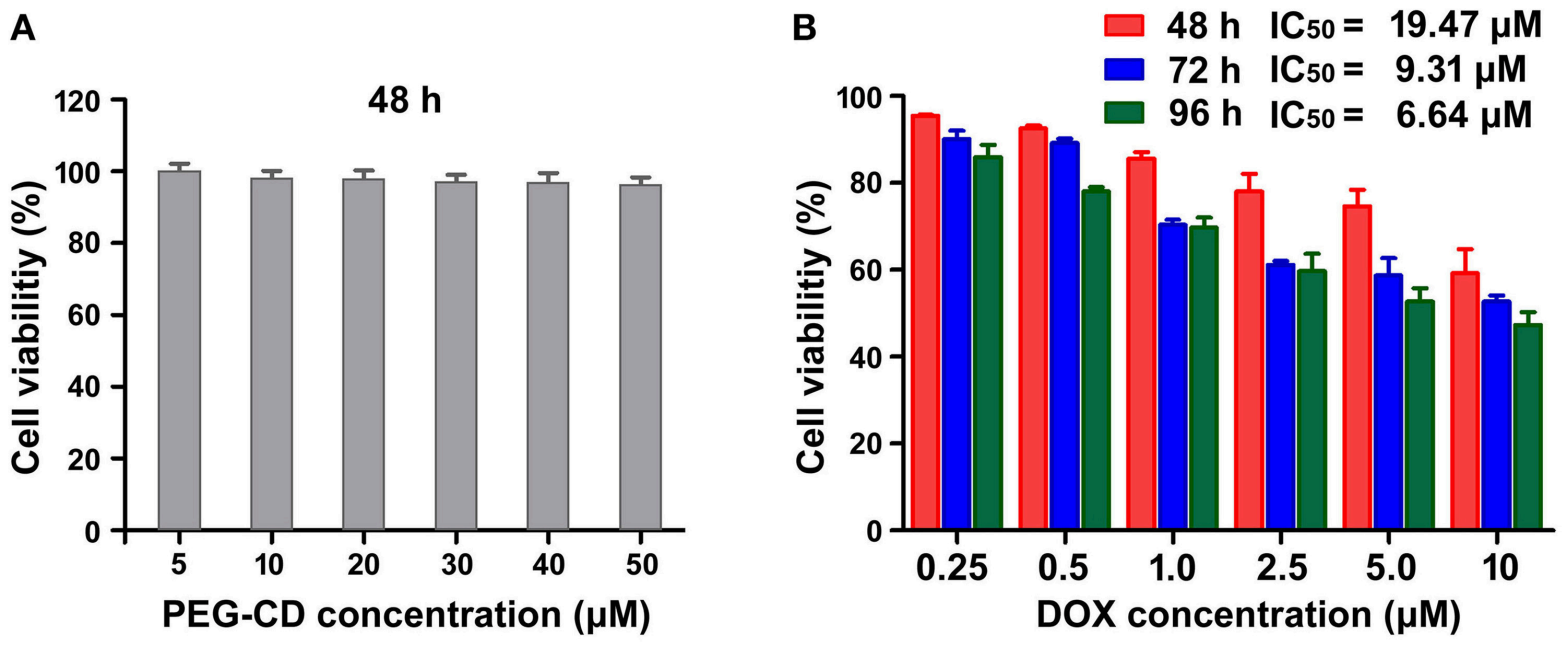
*In vitro* cytotoxicities of PEG-CD **(A)** and PEG-CD/AD nanoparticles **(B)** against HepG2 cells at different concentrations. Data are means ± standard deviation for three separate experiments.

The *in vitro* cytotoxicity of PEG-CD/AD nanoparticles against HepG2 cells was evaluated by CCK-8 agents. Firstly, we determined the inhibitory effect of PEG-CD on the growth of the cells and no significant cytotoxicity was detected since the cell viabilities were larger than 95% at the concentration range of 5–50 μM (Figure 5A). By comparison, PEG-CD/AD nanoparticles exhibited obvious inhibition on cell growth in time- and dose-dependent manners (Figure 5B). The half maximal inhibitory concentrations (IC_50_ values), denoted as the concentration of DOX causing 50% of cell growth, were calculated to be 19.47, 9.31, and 6.64 μM for 48, 72, and 96 h, respectively. In addition, PEG-CD/AD nanoparticles showed weaker cytotoxicity in HepG2 cells than that of free DOX (Figure S6). According to other disulfide linked DOX-prodrug reported previously (Song et al., 2016), we deduced that it was because PEG-CD/AD showed a sustained release of DOX and thus exhibited delayed therapeutic effects, while free DOX entered the cell nuclei directly and exerted their therapeutic effects instantly.

### Physicochemical characterization of PEG-CD/AD/SF nanoparticles

**Figure 6:**
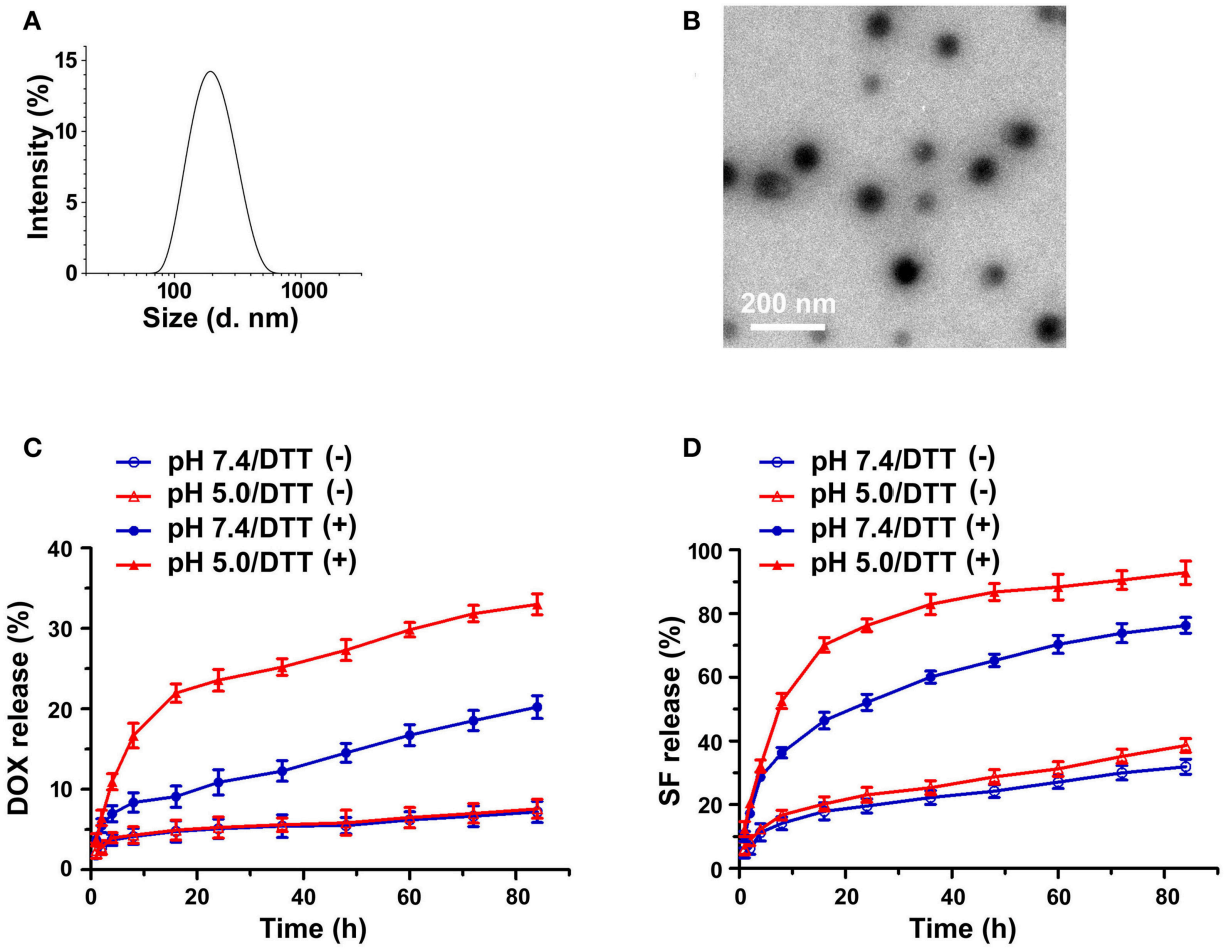
Characterization of PEG-CD/AD/SF nanoparticles. Hydrodynamic diameter distribution **(A)** and TEM image **(B)** of PEG-CD/AD/SF nanoparticles; *in vitro* releases of DOX **(C)**, and SF **(D)** from PEG-CD/AD/SF nanoparticles in different release media. Data are means ± standard deviation for three separate experiments.

Sorafenib (SF) was further integrated into the nanosystem *via* hydrophobic interaction and π-π stacking interaction (Zhao et al., 2015) between DOX and SF. The core of the nanosystem was composed of DOX and SF, while the shell was a layer of PEG-CD. According to a previous study (Zhang et al., 2016), the CI values (combination index determined by IC_50_) in DOX-based combinations (DOX/SF molar ratio: 1/0.1, 1/0.2, 1/0.5) were much lower than that of SF-based combinations (DOX/SF molar ratio: 0.1/1, 0.2/1), indicated that DOX-based combinations present better cytotoxic effect. In our work, the hydrodynamic diameters of PEG-CD/AD/SF nanoparticles at different molar ratios were evaluated and the results are displayed in Table 1. The average size of PEG-CD/AD/SF nanoparticles increased as the molar ratio of PEG-CD: AD: SF changed from 1/1/0.1 to 1/1/2. Meanwhile, the PDI of PEG-CD/AD/SF nanoparticles tended to be large when more SF was added, indicating that excess SF had a negative effect on the stability of PEG-CD/AD/SF nanoparticles. Taken a comprehensive consideration of CI values and sizes, 1/1/0.2 was believed as the optimal ratio to prepare PEG-CD/AD/SF nanoparticles. As shown in Figure 6A, PEG-CD/AD/SF nanoparticles prepared at this ratio had an average size of 186.2 nm with a relatively narrow size distribution (PDI = 0.114). TEM images showed that PEG-CD/AD/SF nanoparticles displayed uniform spherical structure (Figure 6B).

**Table 1:** Characterization of PEG-CD/AD/SF nanoparticles by DLS.

**PEG-CD/AD/SF (molar ratio)**	**1/1/0.1**	**1/1/0.2**	**1/1/0.5**	**1/1/1**	**1/1/2**
Size (nm)	176.1	186.2	202.3	246.4	560.2
PDI	0.096	0.114	0.218	0.306	0.572

The *in vitro* releases of DOX and SF from PEG-CD/AD/SF nanoparticles were investigated in different media and the release profiles are displayed in Figures 6C,D. Obviously, DOX releases from PEG-CD/AD/SF nanoparticles in the media containing DTT were significantly faster than that in the media without DTT. DOX release amounts reached up to 20.2 and 33.0% in 84 h with the presence of DTT at pH 7.4 and pH 5.0, respectively, whereas were less than 10% in the media without DTT at both pH. We believed that the higher DOX release rates in pH 5.0 PBS with DTT was attributed to the reduction sensitive properties of disulfide bond as well as better DOX solubility in slightly acid media. The same trend was also observed in the releases profiles of SF from PEG-CD/AD/SF nanoparticles in different media. However, the overall release rates of SF were much higher than that of DOX at the same condition. For example, SF release amounts were 76.3 and 92.8% in 84 h with the presence of DTT at pH 7.4 and pH 5.0, respectively. This difference could be attributed to their distinct drug-loading modes. SF was incorporated into the nanoparticles by physical π-π stacking interaction and hydrophobic interaction, while DOX was loaded by chemical bond and thus exhibited sustained release property.

### *In vitro* cytotoxicity of PEG-CD/AD/SF nanoparticles against HepG2 cells

**Figure 7:**
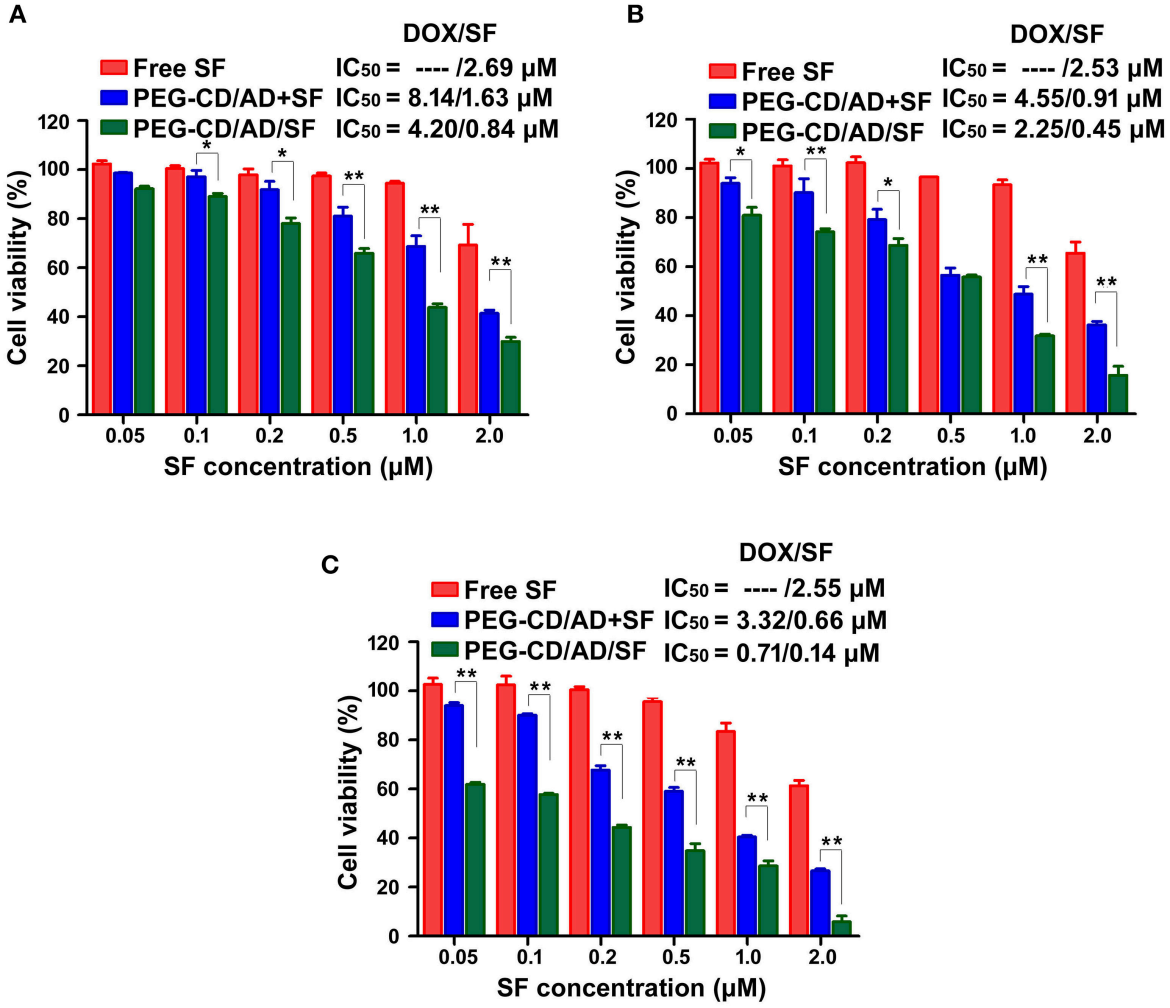
*In vitro* cytotoxicity of HepG2 cells with the treatments of free SF, PEG-CD/AD, and SF physical mixture, PEG-CD/AD/SF nanoparticles for 48 h **(A)**, 72 h **(B)**, and 96 h **(C)**. Data are means ± standard deviation for three separate experiments. *p < 0.05, **p < 0.01.

The cytotoxicity of free SF, PEG-CD/AD+SF physical mixtures and PEG-CD/AD/SF nanoparticles against HepG2 cells were investigated for different time (Figure 7). Compared to PEG-CD/AD nanoparticles (Figure 5B) and free SF, PEG-CD/AD+SF physical mixtures showed an additive cytotoxicity toward HepG2 cells. PEG-CD/AD/SF nanoparticles exhibited higher cytotoxicity than PEG-CD/AD+SF physical mixtures (*p* < 0.05) and the IC_50_ values of PEG-CD/AD/SF nanoparticles were approximately 1.9-, 2.0-, and 4.7-fold decrease compared to that of PEG-CD/AD+ SF physical mixtures after treating HepG2 cells for 48, 72, and 96 h, respectively. The results suggested that PEG-CD/AD/SF nanoparticles exert stronger inhibitory effect against HepG2 cells by co-loading DOX and SF into the same nanosystem. This might perhaps because the solubility of sorafenib greatly improved after loading into the PEG-CD/AD/SF nanoparticles. Moreover, by comparing the IC_50_ values of PEG-CD/AD/SF nanoparticles at different time, we could easily found that the cytotoxicity of PEG-CD/AD/SF nanoparticles increased remarkably as the time increased. This sustained cytotoxicity would be beneficial for treatment of HCC *in vivo*.

## Conclusion

In this study, we successfully synthesized a disulfide linked prodrug of DOX, which could complex with PEG-CD to form supramolecular amphiphiles. PEG-CD/AD amphiphiles could then self-assemble into nanostructures in aqueous solution with a uniform size of 166.4 nm and meanwhile load SF to form PEG-CD/AD/SF nanoparticles with a size of 186.2 nm. PEG-CD/AD nanoparticles possessed reduction-responsive property and could be effectively uptaken by HepG2 cells. The *in vitro* release of DOX and SF from PEG-CD/AD/SF nanoparticles exhibited reduction-responsive manners. Finally, the *in vitro* cytotoxicity assay illustrated that PEG/AD/SF nanoparticles showed two- to five-fold increased cytotoxicity toward HepG2 cells than PEG-CD/AD+SF physical mixtures. Altogether, this reduction-responsive nanoparticle system showed great potential for HCC treatment.

## Author contributions

QX and TS: designed the experiments and wrote the manuscript; MC, JW, and GY: carried out the research and analysis of data and contributed to manuscript writing.

### Conflict of interest statement

The authors declare that the research was conducted in the absence of any commercial or financial relationships that could be construed as a potential conflict of interest. The reviewer YY and handling Editor declared their shared affiliation.
