# Supplementary material for: Facile Fabrication of Reduction-Responsive Supramolecular Nanoassemblies for Co-delivery of Doxorubicin and Sorafenib toward Hepatoma Cells
Source: Front Pharmacol. 2018 Feb 6;9:61. doi: 10.3389/fphar.2018.00061 (PMC5816960; doi:10.3389/fphar.2018.00061)
Supplement: Supplementary file 1 [file Presentation1.pdf]

Supplementary Material

Facile fabrication of reduction-responsive supramolecular nanoassemblies for co-delivery of doxorubicin and sorafenib towards hepatoma cells

Qingqing Xiong, Mangmang Cui, Ge Yu, Jian Wang*, Tianqiang Song*

*** Correspondence:** Dr. Jian Wang: wangjian112358@163.com;

Dr. Tianqiang Song: tjchi@hotmail.com

1 Supplementary Methods

Synthesis of β-cyclodextrin-modified poly(ethylene glycol) (PEG-CD)

Ethyl diamino-β-cyclodextrin (EDA-CD) was synthesized according our previous report. PEG-CD was synthesized by the conjugation of PEG-NHS and EDA-CD according synthesis route displayed in Fig. S1A. Briefly, PEG-NHS (0.3 mmol, 0.6 g) and EDA-CD (0.45 mmol, 0.53 g) were co-dissolved in 0.01 M phosphate buffer saline (pH 4.4) and stirred at RT overnight. Then, the solution was dialyzed for 2 days against deioned water (MWCO 2000) and lyophilized to obtain white product (Yield 40.2%). As shown in Fig. S1B, the integration ratio of the peak at 3.33 ppm, corresponding to the terminated methyl protons of PEG (*signal a*), to the peak at 5.04 ppm, which was attributed to the C1-H of β-cyclodextrin (*signal C1-H*) was 3:7, which indicated that PEG-CD was synthesized successfully.

2 Supplementary Figures

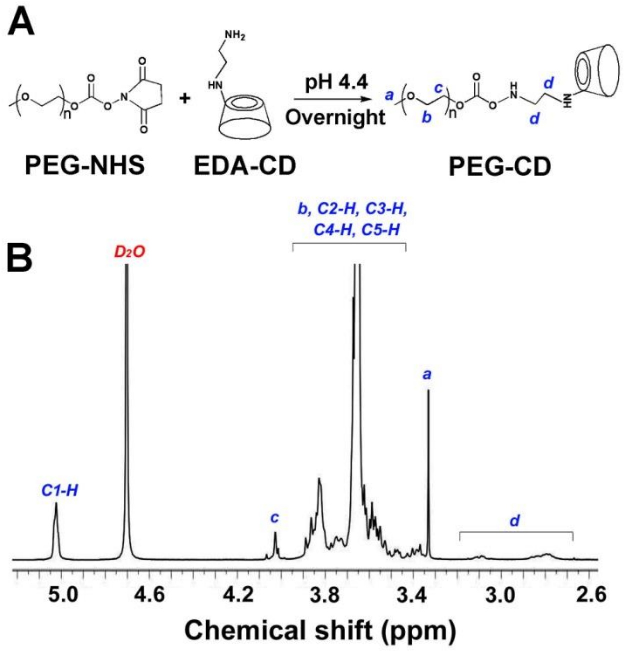

Supplementary Figure 1. Synthetic route (A) and ^{1}H NMR spectra (B) of PEG-CD.

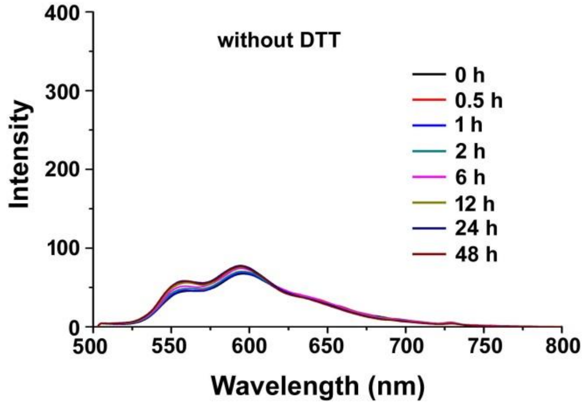

Supplementary Figure 2. Emission spectra of DOX in PEG-CD/AD nanomicelles solution without DTT at different time at an excitation wavelength of 485 nm.

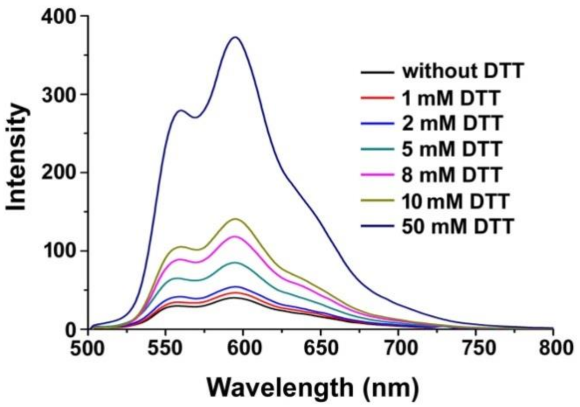

Supplementary Figure 3. Emission spectra of DOX in PEG-CD/AD nanomicelle solutions containing DTT of different concentrations at an excitation wavelength of 485 nm.

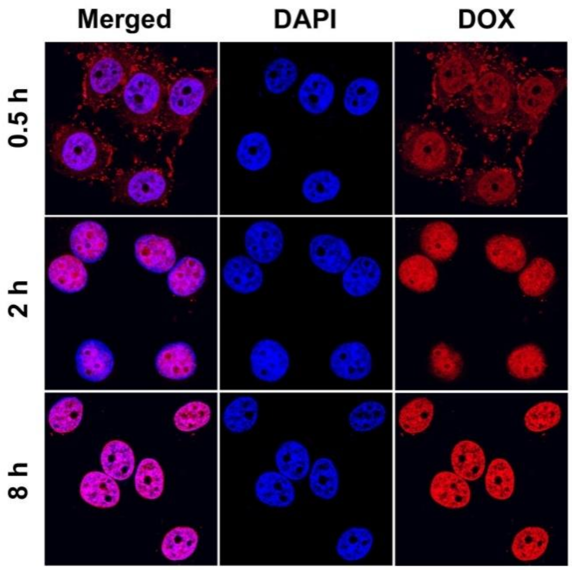

Supplementary Figure 4. Confocal images of the cells with the treatments of free DOX for different time.

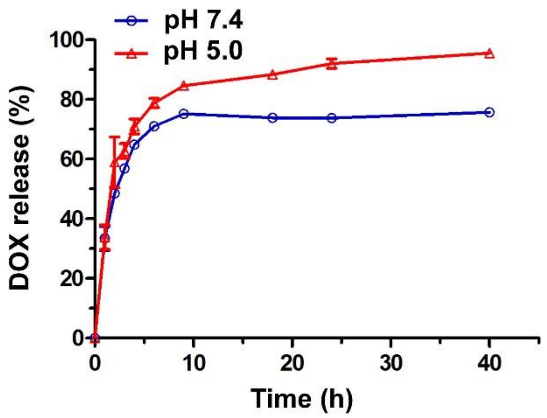

Supplementary Figure 5. *In vitro* release of free DOX in release media with differnent pHs. Data are means±standard deviation for three separate experiments.

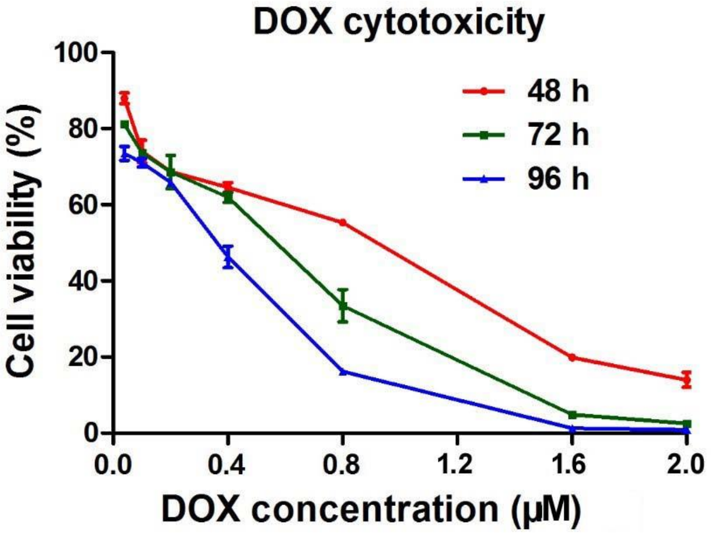

Supplementary Figure 6. *In vitro* cytotoxicity of HepG2 cells with the treatments of free DOX for different time. Data are means±standard deviation for three separate experiments.
